# Supplementary material for: Prevalence and predictors of uterine rupture among Ethiopian women: A systematic review and meta-analysis
Source: PLoS One. 2020 Nov 2;15(11):e0240675. doi: 10.1371/journal.pone.0240675 (PMC7605683; doi:10.1371/journal.pone.0240675)
Supplement: S2 Table — (DOCX) [file pone.0240675.s003.docx]

| **Component** | **Search terms** |
| --- | --- |
| #1 | Uterine Rupture/epidemiology"[Mesh] |
| #2 | uterine rupture"[MeSH Terms] OR ("uterine"[All Fields] |
| #3 | ("rupture"[All Fields]) OR "uterine rupture"[All Fields]) |
| #4 | Ethiopia [Mesh] OR Ethiopia [tiab] |
| #5 | ((#1 AND #2 ) AND #3)) AND #4) or ("Uterine Rupture/epidemiology"[Mesh] OR ("uterine rupture"[MeSH Terms] OR ("uterine"[All Fields] AND "rupture"[All Fields]) OR "uterine rupture"[All Fields])) AND ("ethiopia"[MeSH Terms] OR "ethiopia"[All Fields]) |

S2 Table: PubMed search string of uterine rupture in Ethiopia
