# Supplementary figures and images for: Prevalence and predictors of uterine rupture among Ethiopian women: A systematic review and meta-analysis
Source: PLoS One. 2020 Nov 2;15(11):e0240675. doi: 10.1371/journal.pone.0240675 (PMC7605683; doi:10.1371/journal.pone.0240675)

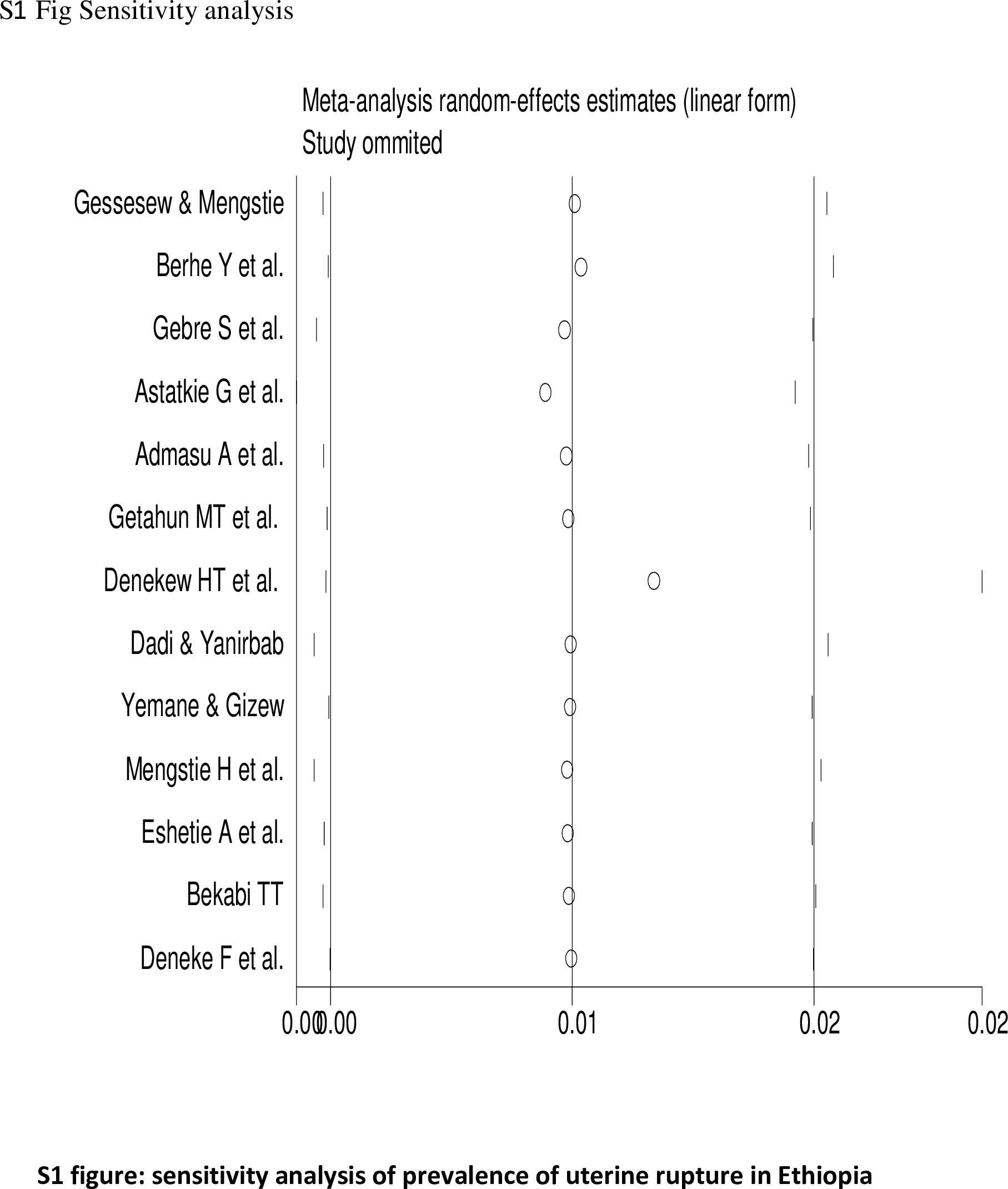

Supplement: S1 Fig — (TIF) [file pone.0240675.s004.tif]
