# Supplementary material for: Prevalence and predictors of uterine rupture among Ethiopian women: A systematic review and meta-analysis
Source: PLoS One. 2020 Nov 2;15(11):e0240675. doi: 10.1371/journal.pone.0240675 (PMC7605683; doi:10.1371/journal.pone.0240675)
Supplement: Attachment — Submitted filename: Response to reviewers.docx [file pone.0240675.s005.docx]

**Dear Editors and reviewers of Plos One**

We would like to extend our deepest appreciation for devoting your time to review our manuscript entitled “Prevalence and predictors of uterine rupture among Ethiopian women: a systematic review and meta- analysis”. Uterine rupture is a global public problem that causes maternal morbidity, which can be prevented through assessing the prevalence and predictors of uterine rupture among women. Even though, the prevalence is inconsistent across the country. Therefore, this systematic review and meta-analysis estimates the pooled prevalence and predictors of uterine rupture among Ethiopian women among women in Ethiopia.

Dear reviewer, there has been a minor revision of the whole manuscript (Abstract, introduction, methods, results, discussion and conclusions) with a correction of the previous edition in clearly manner mainly the points raised by reviewer one. The journal requirements supposed by you have been included as search strategy has included as supplementary file and the whole structure of the manuscript has been revised. We hope now the manuscript is clear and more acceptable than its previous version. We have tried to present the paper in proper manner according to your comment what to supposed to do so. For this, here we have given our responses to each of the concerns you raised, highlighted by red color. Again, we would like to remind our strongest gratitude for your effort for the improvement of this manuscript and the response for each the points were addressed below.

**Regards**

Response for reviewer comments
1) Meta-analyses of proportions are a bit more complicated since transformations are needed to account for the 0 and 100% limits. Step 1: transformation; step 2: meta-analysis method using standard approach (i.e. inverse variance DerSimonian-Laird); step 3: back-transformation to percentages and plotting. One approach is logit transformation. However, a double arcsine transformation is the norm (http://jech.bmj.com/content/early/2013/08/20/jech-2013-203104). The method is implemented in the Stata module metaan http://www.stata-journal.com/article.html?article=st0201. Alternatively, you can manually perform using the command you used (I suspect metan, although not referenced).

Response: Highly valuable comment, I completely agree what you supposed and revision was made. Transformation for meta-analysis is one part of analysis. We did transformations using transformation with a DerSimonian-Laird. Therefore, transformation is typically used using the Statistical packages, of Stata, incorporating the transformations into their software programs for meta analyses of proportions. Because, the proportions contain inadmissible values (<5%) especially when the statistic is near the boundary and computation of confidence intervals is not possible when the statistic is on the boundary, as the estimated standard error is set to zero and as a consequence, the metan command automatically excludes studies with proportion equal to 0 or 1 from the pooled estimate. Thus, what we supposed to informs you is that due to the lower proportions reported in this analysis the figure of the pooled proportion approximates to two digits to the lower one that makes the confidence interval or the confidence limit lowers and even the point estimate and the lower limit sometimes the same due the lower margin of error and lower of the transformed proportion. Hence, margin of error decreases as the sample size increases in our case. For the random effects model, the confidence interval can tend toward zero only with an infinite number of studies (unless the between-study variation is zero). In regard to the citation it is well known that the metaprop command is available on many Cochrane meta-analysis hand books. We have put a citation the main document in the analysis section.
2) Meta-regression is a stab in the dark usually and is underpowered to detect anything but massive associations (effectively a regression with X observations, where X is the number of available studies). You should discuss this as a major limitation. Even with 60 or 80 studies, it can provide little insight.

Response: An insight on the meta-regressions is highlighted in the limitations section. The point estimate I2 should be interpreted cautiously when a meta-analysis has few studies. In small meta-analyses, confidence intervals should supplement or replace the biased point estimate.

3) Cochran Q (i.e. chi-square) is notoriously underpowered to detect heterogeneity, especially for small meta-analyses http://www.ncbi.nlm.nih.gov/pubmed/9595615. I would not use. (see also comment below regarding this and I^2)

Response: accepted and cochran Q is not used in this analysis. It was unfortunately spelling error and excluded and it was already excuded in the modification on the major revisions.
4) Exclude the Getahun study which is an outlier and re-analyse - do things change a lot? Why did the Gretahun study observe a prevalence of 17% and Getahun result is an extreme outlier, some explanation is needed.

Response: **Highly valuable and considered it for sensitivity analysis.** I understand and admits what you suggest on the high prevalence of uterine rupture in Getahun study is 17%. Unfortunately, the Getahun et al finding is a representative and high-quality data that was conducted in the three-referral hospital of Amhara region with a large sample size. But other study setting is only one hospital and also includes district hospitals. Thus, due to being referral hospitals and 3 hospitals with high case flow obstetric complications or maternal near-miss including uterine rupture are expected to be higher than other studies finding. Thus, to know whether the Getahun study finding have influenced the overall the pooled prevalence estimates of uterine rupture we have done a sensitivity analysis. However, the sensitivity analysis that putted as additional file also revealed that the overall pooled prevalence was not affected by the finding of Getahun et al. Furthermore, the study has included almost five years data of the referral hospitals of Amhara regional state, institution-based cross-sectional study, from 2013-2017. Therefore, all authors decided agreed not to exclude this study based on what explained above.

5) Very high heterogeneity estimates which according to some researchers means studies should not be meta-analyzed. However, I disagree with that assessment and I am not surprised at all by your heterogeneity estimates. You have large meta-analyses and there is a direct link between meta-analysis size and detected heterogeneity. I disagree with the argument that when large heterogeneity is picked up, studies should not be combined. In my view, large heterogeneity is the norm and it's great if it has been picked up and can be incorporated in the model. It is much more problematic when the underlying heterogeneity is not picked up and studies are "safely" combined under a homogeneity assumption. I think you can use this to defend your decision and the high heterogeneity:

**Response: Highly valuable and accepted it**

We completely agree with what you supposed to do and did the meta-analysis in this way. We disagree with the suggestions that high heterogeneity estimates mean studies should not be meta-analyzed. Thus, large heterogeneity is the norm and it's great if it has been included and can be incorporated in the model.
6) some editing and language corrections are needed in the methods section such as; report the confidence intervals for I^2 , correct Stata not STATA (not an acronym), from "Besides,..." onwards (which should read as "Next,...")

Response: accepted and revision was made. The overall editing and language corrections has made based on what you supposed to do so through the manuscript. Specifically, the confidence intervals of I^2^ calculated from Stata was incorporated through the documents. STATA was corrected as Stata, besides was replaced by onwards.

7) Which user-written commands in Stata did you use to perform the analyses? please cite.

The metaprop software was used and it was cited in the statistical analysis section.

8) Year may be worth considering in bias assessment, especially if you don't have enough studies for a formal test: http://www.ncbi.nlm.nih.gov/pubmed/25988604. With newer studies we would be more confident.

Response: Accepted, source of bias was done based on year of publication. However, it is not significant source of hetrogentiy and bias.
9) Clarify the weighting for the RR random effects model. Inverse variance (IV) or Mantel Haenszel (MH)? Note that MH is traditionally a fixed effect approach. How was the random-effect model implemented, i.e. how was heterogeneity estimated? There are numerous ways to do so. Did they use the standard DerSimonian-Laird method? If so, please state so. Also, there are better performing methods, for example please see https://www.ncbi.nlm.nih.gov/pubmed/28815652 and the metaan command in Stata where these are implemented.

Response: accepted and revision was made. The details of the statistical methods have been included in the analysis section. Thus, a random effects meta-analysis model was used to estimate the DerSimonian and Laird’s pooled effect. In the current meta-analysis, arcsine-transformed proportions were used. The pooled proportion was estimated by using the back-transform of the inverse variance weight of the transformed proportions, using DerSimonian-Laird weights for the random-effects model. The heterogeneity was also estimated using derSimonian-Laird method.

10) What is ANC visit? Please clear the acronym before the acronym.

Thank you for the comments, we accepted and corrected on the revisions.

11) Authors reported in the introductions “partial rupture in which a defect in the myometrium is covered by the visceral leaf of the peritoneum….it is a dehiscence. Please, state it.

Valuable, Thank you for the concern.

Partial rupture is not mean that uterine dehiscence and some revisions was done. Hence, our outcome of interest was uterine rupture. The word “defect” was replaced by “tear: to show rupture than scar. Up to our knowledge uterine rupture and uterine dehiscence are two different obstetrical terminologies that used to describe uterine injury during delivery. Rupture is often traumatic and may occur in an intact uterus or involve the majority of a uterine scar from previous cesarean delivery. While, dehiscence is a separation that involves only a portion of the uterine scar. Both can be either complete or partial, which complete rupture and dehiscence describe separation of the uterine wall (endometrium, myometrium, and serosa).
12) What was the outcome in the case-control studies? Was it uterine rupture? How can a case-control study provide an estimate of the prevalence of uterine rupture?

**Response:** Thank you for the comments and it was highly valuable**.** The outcome of interest in the included case control studies was uterine rupture. Unfortunately, case control studies didn’t report proportions or prevalence data. Thus, what we did is that we used case control studies in this analysis if these studies reported the total number of cases encountered during the study period and the total number of deliveries reported in that specific period. We didn’t take the sample of cases and controls that used for factor analysis only used by the author. Example, in the study done by Dadi & Yanirbab was a case control study with a participant of 363, but in the results section the total number of cases reported from 2011 to August 2016 was 121 form the total (9789) of women who gave birth in the hospital was used for our analysis and the same for Gebrie S et al a case control study, but report the total number of deliveries (5622) and the total number of cases from 2009 to 2014 in Suhul hospital was 93. But studies which didn’t report such total number of cases and total deliveries in the time period is excluded from the pooled prevalence analysis of uterine rupture such as Abebe F et al, Workie A et al and Bereka TM et al instead of used only for factor analysis.

**Regards**
